# Dietary patterns and asthma among Peruvian children and adolescents

**DOI:** 10.1186/s12890-020-1087-0

**Published:** 2020-03-14

**Authors:** Carla E. Tarazona-Meza, Corrine Hanson, Suzanne L. Pollard, Karina M. Romero Rivero, Rocio M. Galvez Davila, Sameera Talegawkar, Carlos Rojas, Jessica L. Rice, William Checkley, Nadia N. Hansel

**Affiliations:** 1 Center for Non-Communicable Diseases Research and Training, Johns Hopkins University, Baltimore, MD USA; 2 Biomedical Research Unit, Asociacion Benefica PRISMA, Carlos Gonzales 251, San Miguel, Lima, Peru; 3 Division of Medical Nutrition Education, University of Nebraska Medical Center, 984035 Nebraska Medical Center, Omaha, NE 68198-4035 USA; 4 Division of Pulmonary and Critical Care Medicine, Johns Hopkins University, Johns Hopkins Bayview, 1830 E, Monument, 5th Floor, Baltimore, MD 21205 USA; 5 Department of Exercise and Nutrition Sciences, Milken Institute School of Public Health, The George Washington University, 950 New Hampshire Ave, NW. 7th Floor, Washington, DC 20052 USA; 6 Monitoring and Evaluation Office, UNICEF Peru, Parque Meliton Porras No. 350, Miraflores, Lima Peru; 7 Division of Pediatric Pulmonology, School of Medicine, Johns Hopkins University, Baltimore, MD USA

**Keywords:** Asthma, Children, Adolescents, Diet, Peruvian

## Abstract

**Background:**

Asthma is one of the conditions that contributes to the global burden of respiratory diseases and has been previously associated with diet intake. The goal of this study was to determine the relationship between diet, assessed by a developed score, and asthma in Peruvian children.

**Methods:**

This study was a cross sectional analysis nested within an unmatched case-control study of children in two peri-urban communities of Lima, Peru. We evaluated 767 children and adolescents (573 with asthma, 194 controls) between 9 and 19 years. Diet was assessed using a food frequency questionnaire (FFQ), with food groups classified as “healthy” or “unhealthy”. Asthma control, Lung function and atopy were assessed by Asthma Control Test, Spirometry and InmunoCAP 250 test, respectively.

**Results:**

Mean age of participants was 13.8 years (SD 2.6). Mean diet score was 5 (SD 1.23; range 2–8). Healthy Diet Score was associated with asthma status [OR 0.83, 95% CI (0.72, 0.95), *p* = 0.009] in adjusted analysis. Thus, participants with higher HDS, had lower odds of asthma. In sensitivity analyses, when adjusting for atopy, results did not change significantly. [OR 0.85, 95% CI (0.72, 0.99); *p* = 0.04]. No association between the HDS and asthma control, FEV1, nor FeNO were observed. Atopy did not modify the association between diet and asthma outcomes.

**Conclusions:**

In our study cohort, better diet quality was associated with lower odds of asthma, but was not associated with asthma control. Diet modification may be a potential intervention to impact the increasing prevalence of this disease.

## Background

Asthma has been identified as one of the conditions that contribute to the global burden of respiratory diseases [1]. Increasing trends were reported in 2010 in Peru, and approximately 326 deaths and 142,753 disability-adjusted life years were caused by asthma [2]. While factors for the increasing asthma rates are not totally understood, lifestyle and diet may be important contributors [3, 4].

Several studies [5–7] have shown an association between diet intake and asthma symptoms. While intake of nutrient dense foods such as fruits, vegetables, and fish were found to be inversely associated with asthma symptoms and atopy among children and adolescents [5–7]; other caloric dense foods that are high in sugar and fat were associated with an increased prevalence of asthma symptoms [8, 9]. It is possible that the effects of a certain food or nutrient are, in fact, a result of the synergistic effect of multiple diet components, and so investigating a diet pattern in relationship to diseases such as asthma may be more informative [4, 10]. Previous work from our group suggests that adherence to a Mediterranean diet pattern may be associated with lower asthma prevalence in Peruvian children and adolescents [11]; but since the Mediterranean diet score is based on foods that are not frequently consumed in Peru it may not be the most culturally appropriate dietary pattern classification to use in this population.

There are several validated dietary pattern indices and scoring systems available to define the intake of a population, but none have been developed specifically for a pediatric Peruvian population. It is important to characterize the dietary intake of a population using a Food Frequency Questionnaire (FFQ) that lists locally available and culturally appropriate foods. Therefore, the goal of this study was to use a locally prepared food frequency questionnaire, in order to devise a scoring system to represent the degree to which a participant’s diet was consistent with patterns that have been associated with chronic disease. Finally, using our scoring system, we evaluated the relationship between diet pattern and asthma prevalence and control. We hypothesized that diet score that was reflective of overall healthier dietary patterns would be associated with lower odds of having ashma in children and adolescents.

## Methods

### Study setting

This study was conducted in two peri-urban communities south of Lima, Peru: Pampas-San Juan de Miraflores and Villa El Salvador (Sector 1).

### Study design

The present study was a nested unmatched case-control study of children in Peru. Household information in the study setting was provided by the parent Genetics of Asthma Susceptibility to Pollution (GASP) study, which recruited participants from a population census conducted by the Asociacion Benefica PRISMA in 2011.

Of the 666 participants with asthma at baseline in the parent study, all were invited to complete the nutritional assessment (FFQ) and 573 (86%) participated. Of the total 511 participants without asthma in the parent study, only 194 (38%) were randomly selected for this study with a total of 767 participants between 9 and 19 years completing the FFQ. The participants were divided into two groups: asthma and control according to the inclusion criteria.

Participants with asthma were those ever being diagnosed with asthma by a physician, with current asthma-like symptoms and/or use of asthma-medicines in the past 12 months, as previously defined [11, 12]. Participants with a chronic respiratory condition other than asthma, as well as those who were pregnant, were excluded.

Control participants were those who have never been diagnosed by a physician with asthma, have no symptoms, and have normal values during spirometry [13], as previously defined [11].

### Data collection

Questionnaires were interviewer-administered by a trained field staff to participants and their caregivers during home visits. Demographic, comorbidity and medication information were collected at study baseline. Socioeconomic status (SES) score was developed using a Principal Component Analysis using household variables that included household size, parental education and 12 household assets: refrigerator, washing machine, television, computer, internet, cellphone, landline phone, bicycle, car, motorcycle, mototaxi and bus. Thus, the lower the score, the higher levels of poverty.

Participants were administered the validated Spanish-translated questionnaires from the International Study of Asthma and Allergies in Childhood (ISAAC) [11, 14]. These questionnaires collected information about asthma disease, lifestyle characteristics and environmental factors to provide a comprehensive framework of the disease development [14].

Among participants with asthma, the Spanish-validated Asthma Control Test (ACT) questionnaire, was applied [15]. Participants from 9 to 11 years completed the Pediatric Asthma Control test, whereas participants from 12 to 19 years, the adolescent version. The questionnaire assessed asthma symptoms in the last 4 weeks, classifying an ACT score ≤ 19 as uncontrolled asthma. This questionnaire has not been validated in Peruvian settings.

Anthropometric measurements were collected according to international guidelines [16].

Body Mass Index (BMI) was calculated in kg/m^2^ and classified based on standard recommendations for age-and-sex specific body mass index cut-off points, for international children and adolescent populations: underweight, normal, overweight and obese (International Obesity Task Force (IOTF)) [17–19] (Supplementary Material 1).

Atopy status was assessed by a fluorescent enzyme in vitro immunoassay. It was previously described that a positive result (IgE level > 0.1 kUa/L), for any of the three allergen mixes, was considered atopic [11, 20].

A flow-based portable spirometer (SpiroPro, Jaeger/ERT, Hoechberg, Germany) was used to assess lung function in accordance with ATS/ERS guidelines were obtained [11, 21]. We used criteria derived by the Global Lung Health Initiative to obtain predicted values and Z-scores [22].

Fractional Exhaled Nitric Oxide (FeNO) levels were measured in parts per billion (ppb) using a portable chemiluminescence analyzer (NIOXMINO, Aerocrine, Solna, Sweden) according to joint ERS/ATS recommendations [23, 24]. FeNO was measured before spirometric testing. No assessments were made if a participant reported a respiratory infection in the last 2 weeks.

### Dietary assessment

Dietary intake information was measured by an interviewer-administered 170 item FFQ list, based on local and typical food items (Supplementary material 2). Study participants completed the FFQ and were asked how often they had consumed each of the 170 food items listed in the last 14 days; responses included never, 1–2, 3–5, 6–9, 10–13 and 14 or more time (as previously described) [11].

These food items were then classified into “groups” based on similar nutrient content and the grouping criteria used in other dietary pattern scoring systems. Eleven food groups were identified from our FFQ: vegetables, fruits, cereals, sugary drinks, nuts and legumes, meat, processed meat, fried foods and added fat high in saturated fat, fish and seafood, polyunsaturated fats, salt; according to the Alternate Healthy Eating Index, which were previously found associated with chronic diseases [25].

These food groups were classified as “healthy” (vegetables, fruits, cereals nuts and legumes, meat, fish and seafood, polyunsaturated fats) or “unhealthy” (sugary drinks, processed meat, fried foods and added fat high in saturated fat, salt) food groups based on the Alternate Healthy Eating Index (AHEI) [25]. Specifically, foods that contributed in a positively to the AHEI score were classified as “healthy”, and foods that contributed in a negatively to the score were classified as “unhealthy”. Participant’s daily frequency values within food groups were then summed, resulting in a total score for each group. Frequency values for all “healthy” and “unhealthy” food groups were then added together separately obtaining one score for the healthy group, and one score for the unhealthy group. The scores for each of these categories were divided into quartiles. The healthy food group was scored 1 through 4, with 1 being the lowest quartile of daily frequency and 4 being the highest. The unhealthy food group was scored 1 through 4 inversely, with 1 being the highest daily frequency and 4 being the lowest.

The final Healthy Diet Score (HDS) was created by summing together the scores for the healthy and unhealthy food groups for a same participant. A higher score was considered to be representative of a better quality, comprised of foods that have been associated with decreased risk of chronic disease.

Dietary frequencies outliers values were corrected by Winzorization to avoid removing participants from main analysis [26].

### Statistical analysis

Summary statistics for participants characteristics (age, sex, Body Mass Index – BMI, SES, site of residence) according to asthma status, were compared. The HDS score was analyzed as a continuous variable and also as a categorical variable (quartiles and dichotomized above and below the median). Chi-squared tests were used for categorical variables. Depending on the distribution of the continuous variables, T-test and Mann Whitney test were used.

We used logistic regression models to examine the association between HDS and asthma (asthma vs. control). Confounding variables included in the final regression models were those determined a-priori based on preliminary evidence on the association with diet and asthma in the relevant literature and included age, sex, BMI, SES and site of residence. In sensitivity analyses, models were also adjusted for atopic status. In addition, in order to determine whether the association of HDS and asthma status was different between those with and without atopy, an interaction term between HDS with atopic status was added to the model.

Furthermore, in secondary analyses among those with asthma, we also examined associations between HDS and asthma control (controlled = ACT score > 19 vs. uncontrolled = ACT ≤19), FEV1, atopy and FeNO. Multivariate linear regression models were used for continuous outcomes (FEV1 and FeNO).

All analyses were performed using STATA 11 statistical software (Stata Corp., College Station, Texas). Statistical significance was considered at the 0.05 level.

## Results

### Participant characteristics

Data from 767 participants were analyzed, including 573 participants with asthma and 194 healthy controls. Healthy Diet Score was normally distributed, whereas age, BMI, SES score and Asthma Control Test, were not normally distributed. Overall, median age was 13.6 years (IQR 11.62–15.85), and participants with asthma were slightly older than controls (*p* = 0.01). Participants with asthma were also more likely to be male, had a higher mean BMI, higher SES, and were more likely to be atopic (Table 1). Among participants with asthma, 15.2% had uncontrolled asthma at baseline.

**Table 1:** Clinical and demographics characteristics of study participants

	Asthma (*n* = 573)	Control (*n* = 194)	Total (*n* = 767)	*p*- value
Age yr, median (IQR)	13.84 (11.66–16.15)	13.05 (11.50–15.26)	13.59 (11.62–15.85)	0.01^a^
Male, n (%)	323 (56.4%)	89 (45.9%)	412 (53.7%)	0.01
Body Mass Index (kg/m2)	**Asthma (*n*** **= 501)**	**Control (*n*** **= 185)**	**Total (*n*** **= 686)**	< 0.01^a^
median (IQR)	21.87 (19.49–24.46)	20.77 (18.99–22.83)	21.46 (19.25–24.30)	
BMI category				0.03
Underweight / Normal, *n* (%)	292 (58.3%)	125 (67.6%)	417 (60.8%)	
Overweight / Obese, *n* (%)	209 (41.7%)	60 (32.4%)	269 (39.2%)	
SES score, median (IQR)	0.57 (− 0.91 to 1.78)	0.14 (−1.27 to 1.47)	0.36 (−1.01 to 1.72)	0.01^a^
HEALTHY DIET SCORE, (% below median)	188 (32.8%)	45 (23.2%)	233 (30.4%)	0.01
Atopy status	**Asthma (*n*** **= 506)**	**Control (*n*** **= 141)**	**Total (*n*** **= 647)**	
Atopic, *n* (%)	387 (76.5%)	76 (53.9%)	463 (71.6%)	< 0.01

^a^ Mann-Whitney

There were no statistically significant differences in age (13.3 vs. 13.6 years, *p* = 0.15), sex (46.3% vs. 49.1% male, *p* = 0.37) between individuals who completed the FFQ and those who did not (767 randomly selected vs. 1177 parent study). While BMI was higher among participants who completed the FFQ compared to participants who did not (22.0 vs. 21.5, *p* = 0.04); we did not find a statistically significant difference in those completing the FFQ for the sub-groups of participants with asthma (22.2 vs. 22.1, *p* = 0.79) and controls (21.4 vs. 21.3, *p* = 0.84).

### Healthy diet score and asthma status

For all participants, mean HDS was 5.00 (SD + 1.23) and ranged from 2 to 8. There were no statistically significant differences in daily frequencies of intake per specific food group between asthma and control groups (Table 2). However, in the unadjusted analyses, the HDS as a continuous measure was inversely associated with asthma status [OR 0.85, 95% CI (0.75, 0.88), *p* = 0.02] and those with asthma were more likely to fall below the median of HDS score compared to controls (23.2% vs. 32.8%, *p* = 0.01), respectively. After adjustment for confounders, a higher HDS score continued to show a statistically significant association with asthma status, with each 1 unit increase in HDS associated with a 17% reduction in the odds of having asthma [OR 0.83, 95% CI (0.72, 0.95), *p* = 0.009]. Results were similar when dichotomizing HDS above and below the median. After adjustment for confounders, subjects with higher HDS had lower odds of asthma, with those in the higher HDS catetogory having a 39% decrease in the odds of having asthma compared to those in the lower HDS category [OR 0.61, 95% CI (0.41, 0.91), *p* = 0.02] (Table 3).

**Table 2:** Daily frequencies intake of food groups by status, unadjusted analyses

Food groups	Control (*n* = 194)	Asthma (*n* = 573)	*p*-value
Vegetables	8.10	8.07	0.92
Fruits	2.07	2.04	0.89
Cereals and grains	4.69	4.47	0.13
Sugary drinks	0.48	0.44	0.21
Nuts and legumes	1.16	1.15	0.62
Meat	1.21	1.20	0.63
Processed meat	0.34	0.37	0.34
Fried foods and added fat high in sat fat	0.74	0.75	0.97
Fish and Seafood	0.27	0.27	0.62
Polyunsaturated Fats	2.56	2.75	0.06
Salty	2.65	2.74	0.34

**Table 3:** Healthy Diet Score (HDS) and risk of asthma using multivariable models adjusted by confounders

Asthma status	OR	*p*-value	(95% Confidence Interval)	
HDS continuous	0.83	0.009	0.72	0.95
Sex	0.64	0.012	0.45	0.91
Age	1.10	0.007	1.03	1.18
BMI dichotomized	1.53	0.024	1.06	2.22
SES score	1.14	0.023	1.02	1.28
Site	1.10	0.626	0.75	1.62
HDS continuous (adjusted by atopy)	0.85	0.043	0.72	0.99
Sex	0.74	0.140	0.49	1.11
Age	1.10	0.028	1.01	1.19
BMI dichotomized	1.50	0.065	0.98	2.30
SES score	0.64	0.028	1.02	1.33
Site	1.16	0.058	0.40	1.02
Atopy	3.04	0.000	2.00	4.64
HDS dichotomized	0.61	0.015	0.41	0.91
Sex	0.64	0.012	0.45	0.91
Age	1.11	0.006	1.03	1.19
BMI dichotomized	1.54	0.023	1.06	2.23
SES score	1.14	0.024	1.02	1.28
Site	1.07	0.727	0.73	1.58

*OR* Odds Ratio

*CI* 95% Confidence Interval

*BMI* Body Mass Index

*SES* Socioeconomic Status

In sensitivity analyses, when adjusting for atopy, results did not change significantly. HDS continued to be associated with lower odds of asthma, using both the continuous [OR 0.85, 95% CI (0.72, 0.99); *p* = 0.04] and dichotomous [OR 0.55, 95% CI (0.35, 0.87); *p* = 0.01] HDS. Furthermore, atopy did not modify the relationship of HDS and asthma status (p-interaction = 0.23).

### Healthy diet score and markers of asthma morbidity

Among participants with asthma, median asthma control score was 24 (IQR 21–25), median lung function (defined as FEV_1_ z scores), 0.97 (IQR 0.12–1.79) and median fractional exhaled nitric oxide (FeNO), 2.77 (IQR 2.30–3.64).

Multivariate regression models showed that continuous HDS was not associated with markers of disease severity, including asthma control, [OR 1.01, 95% CI (0.82, 1.24), *p* = 0.94] or lung function [β-Coef. 0.02, 95% CI (− 0.08, 0.11) *p* = 0.75]. Furthermore, HDS was also not associated with (FeNO) levels [β-Coef. 0.02, 95% CI (− 0.05, 0.08), *p* = 0.65)], or atopic status [OR 1.09, 95% CI (0.91, 1.31), *p* = 0.34].

## Discussion

In this study, we created a “healthy diet score” specific to the intakes of Peruvian children. This score was specifically developed in order to reduce misclassification of diet quality in participants with specific dietary patterns in this setting, and thus yield reliable and valid information of diet-disease relationships. We found that a higher score, indicating a better quality diet, was associated with a lower odds of having asthma in peri-urban Peruvian children aged 9 to 19 years. These results support a growing body of literature suggesting that dietary intake can have an impact on chronic inflammatory diseases [27, 28].

Several studies have evaluated the impact of dietary patterns on asthma and asthma outcomes in children and adolescents, with the Mediterranean diet pattern being a common approach. Recently, Rice et al. found a relationship between a Mediterranean diet score and asthma status in a subset of the Peruvian population used in this analysis [11]. Similar relationships between a Mediterranean-type diet and asthma have been described in children in other geographic locations, including 6–8 year old Spanish children [29–31] and 6–9 year olds in Mexico [32, 33]. The majority of these studies have been conducted in countries where a Mediaterranean type diet is culturally appropriate. In one study that evaluated a Mediterranean-type diet pattern in non-Mediterranean countries, Nagel et al. evaluated 50,004 school children from 8 to 12 years of age in 29 sites across 20 countries. They found food selection according to a “Mediterranean-type diet” was associated with a lower prevalence of current wheeze and ever asthma [34]. Likewise, Papamichael et al. systematized this information and found that the adherence to Mediterranean diet had a protective effect on asthma symptoms, such as nocturnal coughs, lung function and hospital admissions [35].

However, many cultures do not generally consume meals in accordance with a Mediterranean type diet. In Peruvian children, only a moderate adherence to the Mediterranean diet was reported, thus, there is a need for identification of culturally appropriate dietary patterns with beneifical effects. Nevertheless, our score, based on the food groups on the Alternative Healthy Eating Index, also characterizes fruits, vegetables and whole grains as healthy groups, that may have a protective effect on respiratory diseases [25].

Therefore, in our study, it was of interest to develop a diet quality index which would include foods commonly consumed in Peru, which vary from those typically consumed in countries surrounding the Mediterrean sea. This Alternate Health Eating Index 2010 (AHEI-2010) [25] score represents overall diet quality and also has the advantage of providing a measure of diet that incoroporates nutrient and food interactions that are likely of biological importance in asthma. Inclusion for components in the AHEI are based on evidence for diet-disease relationships in the current literature and the score has been associated with major chronic diseases such heart disease, stroke, diabetes [25] and chronic obstructive pulmonary disease [36]. In addition to the total AHEI score, the individual scoring components (i.e. fruit and vegetable intake) and chronic diseases have been observed in many other populations [25], proving a rationale for our scoring system, which used the AHEI categories as a reference but included specific and unique foods commonly consumed by Peruvian children. Additionally, although there are many similarites between the two diet patterns scores, the HDS provides a mechanism for substracting points for “unhealthy foods”, such as sugar sweetened beverages and fast foods, where as the MDS assigned points for intakes of foods consisent with a Mediterranean diet pattern scores.

In addition to a Mediterrean-type diet, other diet patterns have been evaluted in children in conjunction with asthma status. Recently, Poongadan et al. found that individuals with asthma (age 6–40 years) in India had a dietary pattern that was significantly higher in fast foods, salted snacks, fried snacks, and nuts and dried fruit when compared to controls (p<0.05). They also had a higher consumption of sugar and carbonated beverages [37]. Lin et al. (2016) used a factor analysis approach to show that children eating a “healthy” diet had lower rates of wheezing, allergic rhinitis, and bronchitis when compared to those on a high protein, high fat “Western” diet; however, this association was no longer significant after adjustment for confounders [38]. Lee et al. used reduced rank regression methodology to identify a dietary pattern characterized by high consumption of fast foods, high-fat snacks, candy and cookies, and low consumption of fruit, vegetables and rice that was associated with increased risk of current and severe asthma [9]. While these studies used slightly different methodology in the assessment and derivation of dietary patterns, they mostly support an association between lower consumption of nutrient dense foods such as fruits, vegetables, whole grains, and polyunsaturated fatty acids, and the risk for asthma and adverse respiratory events increases in children. We now confirm this finding in a population of children from South America, which may have unique dietary patterns that differ from both the Western diet and Mediterranan diet, due to cultural, environmental, and geographical factors.

In the present study, diet was not associated with worse asthma control, lung function, FeNO or atopy among participants with asthma. This is consistent with other studies where an association between diet and asthma case status was identified, but not an association between diet and the severity of asthma. For example, Rice et al. also reported an association between diet and a lower risk of disease but did not show a statistically significant association between diet and asthma severity [11]. Additionally Han et al. showed that diet was not associated with asthma exacerbations, however, participants with unhealthy diet and vitamin D deficiency were more likely to have severe asthma [39].

Furthermore, exhaled Nitric Oxide (eNO) has been described as an eosinophilic marker [23]. Thus, the finding that HDS is not associatd with eNO or atopic status, and adjusting for atopy in the primary analysis does not significantly change the association of HDS with asthma stuatus, suggest that the link between healthy diet and asthma status may be independent of effects on allergic status or eosinophilic inflammation. Our results corroborate a study conducted in school age children in Mexico City, where Romieu et al. did not find any significant association between Mediterranean diet Index and eNO among children with asthma [33]. However, a recent Randomized Control Trial, a supplementation of 150 g cooked fatty fish in two meals per week, showed a significant reduction of bronchial inflammation after a 6-month intervention. FeNO values reduced on 14.15 ppb in comparison to those that were not supplemented [40].

There are several mechanisms which have been proposed through which diet could play a role in asthma development. A low antioxidant dietary intake, indicated by low intake of fruits and vegetables, can increase oxidative damage of airways by reactive oxygen species generation. Oxidative stress plays a role in the pathogenesis of asthma, and dietary intake of anti-oxidants improves anti-oxidant defenses [41]. Diets low in polyunsaturated fatty acids and omega-3 fatty acids have been linked to asthma as well, possibly due to the anti-inflammatory role of omega-3 fatty acids [42]. Additionally, fruits and vegetables as well as whole grains high in fiber could exert anti-inflammatory effects due to the production of short chain fatty acids, including butyrate, by microbiota in the gut through the fermentation of fiber [43]. Although the relationship between fiber intake and respiratory outcomes has been more commonly conducted in chronic obstructive pulmonary disease [44, 45], there are studies evaluating fiber intake in asthma. Berthon et al. showed that fiber intake was 5 g a day less in asthmatics when compared to controls, and fiber intake in asthmatics was positively associated with lung function [46]. However, mechanisms for these association have not been fully elucidated. Recent evidence has also provided new insight into epigenetic factors that could be associated with respiratory health outcomes in children. A 2017 study by Montrose et al. found that the methylation of the IFNy promoter CpG site-186 was associated with the intake of certain nutrients (including folate and choline), providing evidence that dietary intake of certain foods, such as fruits, vegetables and whole grains, particulary those that function as methyl donors, could be important in epigenetic regulation as it pertains to asthma [47].

Limitations of our study include the cross-sectional design, which limits our ability to study casual relationships. Additionally, recall bias may impact dietary data collected via a FFQ as well as other potential confounders, such as parental allergy and second hand smoking not included in the analyses. Finally, this FFQ has not been validated in our study population yet, which limited, mainly, the interpretation of our results to only frequencies and did not allow us to present results in quantity, such as energy and macronutrients. Our study however, has several strengths. First, it is advantageous to study the relationship between overall dietary patterns and asthma, since no one indivuidal dietary factor consistently stands out as a risk factor. Second, we used a scoring system based on population information that included local, culturally accepted foods that are most likely to be consumed by the population of Peruvian children, who are at high risk for asthma and other studies used similar methodologies using FFQs [35]. Our sample size is large and representative of peri-urban dwelling Peruvian children. Associations remained significant after adjustment for multiple confounders including BMI, which has signicantly attenuated the results of other lifestyle scoring systems [48].

## Conclusions

Our findings support the importance of healthy diets in multi-interventional approaches to prevent asthma. Dietary intake may represent a modifiable environmental exposure that could be targeted to alleviate the current burden of asthma in Peruvian children.

## Supplementary information

**Additional file 1: Supplementary Material 1.** BMI Classification (International Obesity Task Force, IOTF). **Supplementary Material 2.** Food Frequency Questionnaire – English version.

## Data Availability

The datasets used and/or analysed during the current study are available from the corresponding author on reasonable request.

## Abbreviations

ACT: Asthma Control Test
ATS: American Thoracic Society
BMI: Body Mass Index
ERS: European Respiratory Society
FeNO: Fractional Exhaled Nitric Oxide
FEV_1_: Forced expiratory volume in one second
FFQ: Food Frequency Questionnaire
FVC: Forced Vital Capacity
HDS: Healthy Diet Score
ISAAC: International Study of Asthma and Allergies in Childhood
SES: Socio-Economic Status

## References

[1] Ferkol T, Schraufnagel D (2014). The global burden of respiratory disease. Ann Am Thorac Soc.

[2] Institute for Health Metrics and Evaluation (IHME) (2014). GBD database.

[3] Global asthma network. The global asthma report 2014. Auckland. http://www.globalasthmareport.org/; 2014.

[4] McKeever TM, Britton J (2004). Diet and asthma. Am J Respir Crit Care Med.

[5] Chatzi L, Apostolaki G, Bibakis I (2007). Protective effect of fruits, vegetables and the mediterranean diet on asthma and allergies among children in Crete. Thorax..

[6] Chatzi L, Torrent M, Romieu I (2007). Diet, wheeze, and atopy in school children in menorca, Spain. Pediatr Allergy Immunol.

[7] D'Innocenzo S, Matos SM, Prado MS (2014). Dietary pattern, asthma, and atopic and non-atopic wheezing in children and adolescents: SCAALA study, Salvador, Bahia State, Brazil. Cad Saude Publica.

[8] Huang SL, Pan WH (2001). Dietary fats and asthma in teenagers: analyses of the first nutrition and health survey in Taiwan (NAHSIT). Clin Exp Allergy.

[9] Lee SC, Yang YH, Chuang SY, Liu SC, Yang HC, Pan WH (2012). Risk of asthma associated with energy-dense but nutrient-poor dietary pattern in taiwanese children. Asia Pac J Clin Nutr.

[10] Waijers PM, Feskens EJ, Ocke MC (2007). A critical review of predefined diet quality scores. Br J Nutr.

[11] Rice JL, Romero KM, Galvez Davila RM (2015). Association between adherence to the mediterranean diet and asthma in Peruvian children. Lung..

[12] Lai CK, Beasley R, Foliaki S, Shah J, Weiland S, International Study of Asthma and Allergies in Childhood Phase Three Study Group (2009). Global variation in the prevalence and severity of asthma symptoms: phase three of the international study of asthma and allergies in childhood (ISAAC). Thorax.

[13] National Institutes of Health, National Heart, Lung and Blood Institute (2007). National Asthma Education and Prevention Porgram: Expert Panel Report 3: Guidelines for the Diagnosis and Maagement of Asthma.

[14] Asher MI, Keil U, Anderson HR (1995). International study of asthma and allergies in childhood (ISAAC): rationale and methods. Eur Respir J.

[15] Nathan RA, Sorkness CA, Kosinski M (2004). Development of the asthma control test: a survey for assessing asthma control. J Allergy Clin Immunol.

[16] NHANES (2007). anthropometry procedures manual.

[17] Cole TJ, Lobstein T (2012). Extended international (IOTF) body mass index cut-offs for thinness, overweight and obesity. Pediatr Obes.

[18] Cole TJ, Flegal KM, Nicholls D, Jackson AA (2007). Body mass index cut offs to define thinness in children and adolescents: international survey. BMJ..

[19] Cole TJ, Bellizzi MC, Flegal KM, Dietz WH (2000). Establishing a standard definition for child overweight and obesity worldwide: international survey. BMJ..

[20] ImmunoCAP250; Phadia, Kalamazoo, Michigan, USA. Available at: https://www.thermofisher.com/diagnostic-education/lab/wo/en/tests-systems/systems/phadia-250-instrument.html.

[21] Miller MR, Hankinson J, Brusasco V (2005). Standardisation of spirometry. Eur Respir J.

[22] Quanjer PH, Stanojevic S, Cole TJ (2012). Multi-ethnic reference values for spirometry for the 3-95-yr age range: the global lung function 2012 equations. Eur Respir J.

[23] Dweik RA, Boggs PB, Erzurum SC (2011). An official ATS clinical practice guideline: interpretation of exhaled nitric oxide levels (FENO) for clinical applications. Am J Respir Crit Care Med.

[24] Reddel HK, Taylor DR, Bateman ED (2009). An official American Thoracic Society/European Respiratory Society statement: asthma control and exacerbations: standardizing endpoints for clinical asthma trials and clinical practice. Am J Respir Crit Care Med.

[25] Chiuve SE, Fung TT, Rimm EB (2012). Alternative dietary indices both strongly predict risk of chronic disease. J Nutr.

[26] Rompay M, McKeown N, Castaneda-Sceppa C, Falcón L, Ordovás J, Tucker K (2014). Acculturation and sociocultural influences on dietary intake and health status among puerto rican adults in Massachusetts. J Acad Nutr Diet.

[27] Agudo A, Cayssials V, Bonet C (2018). Inflammatory potential of the diet and risk of gastric cancer in the european prospective investigation into cancer and nutrition (EPIC) study. Am J Clin Nutr.

[28] Maijo M, Clements SJ, Ivory K, Nicoletti C, Carding SR (2014). Nutrition, diet and immunosenescence. Mech Ageing Dev.

[29] Garcia-Marcos L, Canflanca IM, Garrido JB (2007). Relationship of asthma and rhinoconjunctivitis with obesity, exercise and mediterranean diet in spanish schoolchildren. Thorax..

[30] Chatzi L, Torrent M, Romieu I (2008). Mediterranean diet in pregnancy is protective for wheeze and atopy in childhood. Thorax..

[31] Castro-Rodriguez JA, Garcia-Marcos L, Alfonseda Rojas JD, Valverde-Molina J, Sanchez-Solis M (2008). Mediterranean diet as a protective factor for wheezing in preschool children. J Pediatr.

[32] de Batlle J, Garcia-Aymerich J, Barraza-Villarreal A, Anto JM, Romieu I (2008). Mediterranean diet is associated with reduced asthma and rhinitis in mexican children. Allergy..

[33] Romieu I, Barraza-Villarreal A, Escamilla-Nunez C (2009). Dietary intake, lung function and airway inflammation in mexico city school children exposed to air pollutants. Respir Res.

[34] Nagel G, Weinmayr G, Kleiner A, Garcia-Marcos L, Strachan DP, ISAAC Phase Two Study Group (2010). Effect of diet on asthma and allergic sensitisation in the international study on allergies and asthma in childhood (ISAAC) phase two. Thorax..

[35] Papamichael MM, Itsiopoulos C, Susanto NH, Erbas B (2017). Does adherence to the Mediterranean dietary pattern reduce asthma symptoms in children? A systematic review of observational studies. Public Health Nutr.

[36] Varraso R, Chiuve SE, Fung TT (2015). Alternate healthy eating index 2010 and risk of chronic obstructive pulmonary disease among US women and men: prospective study. BMJ..

[37] Poongadan MN, Gupta N, Kumar R (2016). Dietary pattern and asthma in India. Pneumonol Alergol Pol.

[38] Lin YP, Kao YC, Pan WH, Yang YH, Chen YC, Lee YL (2016). Associations between respiratory diseases and dietary patterns derived by factor analysis and reduced rank regression. Ann Nutr Metab.

[39] Han YY, Forno E, Alvarez M, Colón-Semidey A, Acosta-Perez E, Canino G, Celedón JC (2017). Diet, lung function and asthma exacerbations in Puerto Rican children. Pediatr Allergy Immunol Pulmonol.

[40] Papamichael MM, Katsardis C, Lambert K, Tsoukalas D, Koutsillieris M, Erbas B, Itsiopoulos C (2019). Efficacy of a Mediterranean diet supplemented with fatty fish in ameliorating inflammation in paediatric asthma: a randomized control trial. J Hum Nutr Diet.

[41] Wood LG, Gibson PG (2009). Dietary factors lead to innate immune activation in asthma. Pharmacol Ther.

[42] Mickleborough TD, Lindley MR (2013). Omega-3 fatty acids: a potential future treatment for asthma?. Expert Rev Respir Med.

[43] Trompette A, Gollwitzer ES, Yadava K (2014). Gut microbiota metabolism of dietary fiber influences allergic airway disease and hematopoiesis. Nat Med.

[44] Hanson C, Lyden E, Rennard S (2016). The relationship between dietary fiber intake and lung function in NHANES. Ann Am Thorac Soc.

[45] Varraso R, Willett WC, Camargo CA (2010). Prospective study of dietary fiber and risk of chronic obstructive pulmonary disease among US women and men. Am J Epidemiol.

[46] Berthon BS, Macdonald-Wicks LK, Gibson PG, Wood LG (2013). Investigation of the association between dietary intake, disease severity and airway inflammation in asthma. Respirology..

[47] Montrose L, Ward TJ, Semmens EO, Cho YH, Brown B, Noonan CW (2017). Dietary intake is associated with respiratory health outcomes and DNA methylation in children with asthma. Allergy Asthma Clin Immunol.

[48] Papoutsakis Constantina, Papadakou Eleni, Chondronikola Maria, Antonogeorgos Georgios, Matziou Vasiliki, Drakouli Maria, Konstantaki Evanthia, Priftis Kostas N. (2017). An obesity-preventive lifestyle score is negatively associated with pediatric asthma. European Journal of Nutrition.

