# Supplementary material for: Dietary patterns and asthma among Peruvian children and adolescents
Source: BMC Pulm Med. 2020 Mar 14;20:63. doi: 10.1186/s12890-020-1087-0 (PMC7071620; doi:10.1186/s12890-020-1087-0)
Supplement: Supplementary file 1 — Additional file 1: Supplementary Material 1. BMI Classification (International Obesity Task Force, IOTF). Supplementary Material 2. Food Frequency Questionnaire – English version. [file 12890_2020_1087_MOESM1_ESM.docx]

**Supplementary Material 1:**

**BMI Classification (**International Obesity Task Force, IOTF)

| Age | Underweight | | Normal | | Overweight | | Obesity | |
| --- | --- | --- | --- | --- | --- | --- | --- | --- |
|  | Boys | Girls | Boys | Girls | Boys | Girls | Boys | Girls |
| 9.0 | <13.24 | <13.18 | 13.24 - <19.10 | 13.18 - <19.07 | >=19.10 - <22.77 | >=19.07 - <22.81 | >=22.77 | >=22.81 |
| 9.5 | <13.34 | <13.29 | 13.34 - <19.46 | 13.29 - <19.45 | >=19.46 - <23.99 | >=19.45 - <23.46 | >=23.39 | >=23.46 |
| 10.0 | <13.45 | <13.43 | 13.45 - <19.84 | 13.43 - <19.86 | >=19.84 - <24.00 | >=19.86 - <24.11 | >=24.00 | >=24.11 |
| 10.5 | <13.58 | <13.59 | 13.58 - <20.20 | 13.59 - <20.29 | >=20.20 - <24.57 | >=20.29 - <24.77 | >=24.57 | >=24.77 |
| 11.0 | <13.72 | <13.79 | 13.72 - <20.55 | 13.79 - <20.74 | >=20.55 - <25.10 | >=20.74 - <25.42 | >=25.10 | >=25.42 |
| 11.5 | <13.87 | <14.01 | 13.87 - <20.89 | 14.01 - <21.20 | >=20.89 - <25.58 | >=21.20 - <26.05 | >=25.58 | >=26.05 |
| 12.0 | <14.05 | <14.28 | 14.05 - <21.22 | 14.28 - <21.68 | >=21.22 - <26.02 | >=21.68 - <26.67 | >=26.02 | >=26.67 |
| 12.5 | <14.25 | <14.56 | 14.25 - <21.56 | 14.56 - <22.14 | >=21.56 - <26.43 | >=22.14 - <27.24 | >=26.43 | >=27.24 |
| 13.0 | <14.48 | <14.85 | 14.48 - <21.91 | 14.85 - <22.58 | >=21.91 - <26.84 | >=22.58 - <27.76 | >=26.84 | >=27.76 |
| 13.5 | <14.74 | <15.14 | 14.74 - <22.27 | 15.14 - <22.98 | >=22.27 - <27.25 | >=22.98 - <28.20 | >=27.25 | >=28.20 |
| 14.0 | <15.01 | <15.43 | 15.01 - <22.62 | 15.43 - <23.34 | >=22.62 - <27.63 | >=23.34 - <28.57 | >=27.63 | >=28.57 |
| 14.5 | <15.28 | <15.72 | 15.28 - <22.96 | 15.72 - <23.66 | >=22.96 - <27.98 | >=23.66 - <28.87 | >=27.98 | >=28.87 |
| 15.0 | <15.55 | <15.98 | 15.55 - <23.29 | 15.98 - <23.94 | >=23.29 - <28.30 | >=23.94 - <29.11 | >=28.30 | >=29.11 |
| 15.5 | <15.82 | <16.22 | 15.82 - <23.60 | 16.22 - <24.17 | >=23.60 - <28.60 | >=24.17 - <29.29 | >=28.60 | >=29.29 |
| 16.0 | <16.08 | <16.44 | 16.08 - <23.90 | 16.44 - <24.37 | >=23.90 - <28.88 | >=24.37 - <29.43 | >=28.88 | >=29.43 |
| 16.5 | <16.34 | <16.62 | 16.34 - <24.19 | 16.62 - <24.54 | >=24.19 - <29.14 | >=24.54 - <29.56 | >=29.14 | >=29.56 |
| 17.0 | <16.58 | <16.77 | 16.58 - <24.46 | 16.77 - <24.70 | >=24.46 - <29.41 | >=24.70 - <29.69 | >=29.41 | >=29.69 |
| 17.5 | <16.80 | <16.89 | 16.80 - <24.73 | 16.89 - <24.85 | >=24.73 - <29.70 | >=24.85 - <29.84 | >=29.70 | >=29.84 |
| >=18 | <17.00 | <17.00 | 17 - <25 | 17 - <25 | >=25 - <30 | >=25 - <30 | >=30 | >=30 |

**Supplementary Material 2:**

**Food Frequency Questionnaire – English version**

| **FOOD FREQUENCY QUESTIONNAIRE (FFQ)** | | | |
| --- | --- | --- | --- |
| 1 | Date of visit | 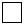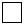-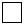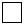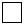-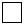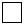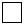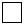 | DD-MMM-AAAA |
| 2 | Date of birth | 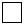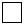-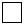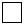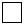-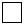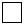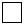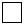 | DD-MMM-AAAA |
| 3 | Address | 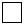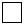 - 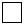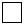- 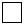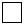- 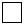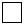 | AAHH/Grupo-Mz-Lt |
| 4 | Sex | 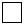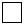 | 01= MALE  02= FEMALE |
| 5 | FFQ # | 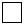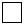 | 01: First Round  02: Second Round |

| **NUM** | **Food Group** | **FOOD ITEMS** | **In the last 2 weeks** (14 days),  **How often have you eaten this food item?** | | | |
| --- | --- | --- | --- | --- | --- | --- |
|  |  |  |  |  |  |  |
|  |  |  | Frequency | | Repetition | |
| 6 | MEAT | Chicken (In casserole, stew, pickled, shredded). | 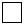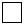 | 01= Never  02= 1-2 times  03= 3-5 times  04= 6-9 times  05= 10-13 times  ----------------------  06= Once a day /  from 14 to 18 times  07= Twice a day /  from 19 to 28 times  08= 3 times a day /  from 29 to 42 times  09= 4 or more times a day /  From 43 to more times | 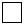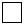 | Fill ONLY if Frequency answer is between 01 and 05:    01= Repeat answer (lunch + dinner)  02= No repetition |
| 7 |  | Chicken , Fried, deep- fried. | 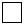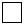 |  | 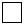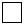 |  |
| 8 |  | Turkey, any preparation | 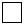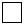 |  | 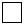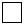 |  |
| 9 |  | Beef , In casserole, stew, ground beef | 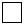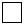 |  | 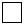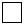 |  |
| 10 |  | Beef , steak, fried | 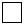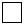 |  | 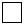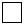 |  |
| 11 |  | Pork, In casserole, stew | 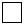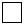 |  | 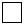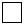 |  |
| 12 |  | Pork, fried,deep- fried | 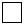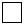 |  | 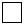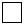 |  |
| 13 |  | Chicken or cow liver, heart, kidneys, chicken guizzard | 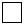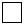 |  | 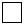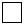 |  |
| 14 | FISH | Fresh blue fish: Mackerel (*Trachurus murphyi*), Pacific Bonito (*Sarda chiliensis)*, anchovy, sardine, tuna, in casserole, stew, pickled, ceviche (raw fish marinated in citrus sauce), tiradito (citrus sauce spiced fish carpaccio) | 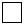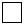 |  | 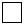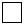 |  |
| 15 |  | Blue fish, fried, deep-fried. | 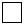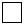 |  | 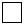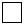 |  |
| 16 |  | Fresh White fish: trout, sea bass in casserole, stew, pickled, ceviche (raw fish marinated in citrus sauce), tiradito (citrus sauce marinated fish carpaccio) | 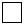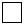 |  | 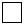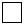 |  |
| 17 |  | White fish, fried, deep-fried. | 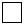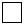 |  |  |  |
| 18 |  | Canned fish (tuna, sardines, anchovies) | 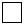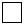 |  |  |  |
| 19 | SEAFOOD | Squid | 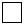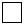 |  |  |  |
| 20 |  | Crab | 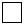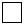 |  |  |  |

| 21 | FATS | Vegetable oil for stews, soups (not for frying) | 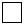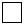 | 01= Never  02= 1-2 times  03= 3-5 times  04= 6-9 times  05= 10-13 times  ----------------------  06= Once a day /  from 14 to 18 times  07= Twice a day /  from 19 to 28 times  08= 3 times a day /  from 29 to 42 times  09= 4 or more times a day /  From 43 to more times |  | Fill ONLY if Frequency answer is between 01 and 05:    01= Repeat answer (lunch + dinner)  02= No repetition |
| --- | --- | --- | --- | --- | --- | --- |
| 22 |  | Vegetable oil for frying | 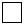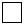 |  |  |  |
| 23 |  | Raw vegetable oil (salads, homemade mayonnaise, other recipes) | 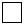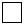 |  |  |  |
| 24 |  | Margarine, any brand (Dorina, La Danesa, Manty, Sello de Oro) | 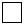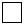 |  |  |  |
| 25 |  | Avocado | 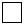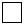 |  |  |  |
| 26 |  | Olives | 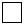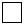 |  |  |  |
| 27 |  | Raisins, pecans, dry plums | 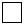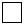 |  |  |  |
| 28 |  | Peanuts | 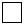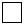 |  |  |  |
| 29 |  | Coco nut (white pulp) raw or as ingredient | 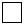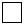 |  |  |  |
| 30 | DELI MEAT | Bologna, Mortadella, Ham |  |  |  |  |
| 31 |  | Hot Dog |  |  |  |  |
| 32 |  | Hamburger, breaded chicken or beef |  |  |  |  |
| 33 |  | Bacon |  |  |  |  |
| 34 |  | Stuffing, “Huacho” style sausage |  |  |  |  |
| 35 |  | Sausage |  |  |  |  |
| 36 | FRUITS | Banana |  | 01= Never  02= 1-2 times  03= 3-5 times  04= 6-9 times  05= 10-13 times  ----------------------  06= Once a day /  from 14 to 18 times  07= Twice a day /  from 19 to 28 times  08= 3 times a day /  from 29 to 42 times  09= 4 or more times a day /  From 43 to more times |  | Fill ONLY if Frequency answer is between 01 and 05:    01= Repeat answer (lunch + dinner)  02= No repetition |
| 37 |  | Orange |  |  |  |  |
| 38 |  | Natural Orange Juice |  |  |  |  |
| 39 |  | Apple |  |  |  |  |
| 40 |  | Papaya |  |  |  |  |
| 41 |  | Pineapple |  |  |  |  |
| 42 |  | Mandarin |  |  |  |  |
| 43 |  | Peach |  |  |  |  |
| 44 |  | Strawberry |  |  |  |  |
| 45 |  | Lucuma |  |  |  |  |
| 46 |  | Mango |  |  |  |  |
| 47 |  | Melon |  |  |  |  |
| 48 |  | Maracuya (Passion Fruit) |  |  |  |  |
| 49 |  | Carambola |  |  |  |  |
| 50 |  | Grapes |  |  |  |  |
| 51 |  | Fruits WITH their skin |  |  |  |  |

| 52 | VEGETABLES | Onion, big pieces |  | 01= Never  02= 1-2 times  03= 3-5 times  04= 6-9 times  05= 10-13 times  ----------------------  06= Once a day /  from 14 to 18 times  07= Twice a day /  from 19 to 28 times  08= 3 times a day /  from 29 to 42 times  09= 4 or more times a day /  From 43 to more times |  | Fill ONLY if Frequency answer is between 01 and 05:    01= Repeat answer (lunch + dinner)  02= No repetition |
| --- | --- | --- | --- | --- | --- | --- |
| 53 |  | Onion as food seasoning |  |  |  |  |
| 54 |  | Raw Carrots (in salads, juice) |  |  |  |  |
| 55 |  | Cooked Carrots (boiled, in soup, stew, spaghetti sauce, as food seasoning) |  |  |  |  |
| 56 |  | Garlic (as food seasoning) |  |  |  |  |
| 57 |  | Raw tomato |  |  |  |  |
| 58 |  | Cooked Tomatoes (Sauté, homemade sauces, dressings) |  |  |  |  |
| 59 |  | Lemon for meals(salads, ceviche, homemade mayonnaise) |  |  |  |  |
| 60 |  | yellow pepper (as dip sauce or as a recipe ingredient) |  |  |  |  |
| 61 |  | Red Hot chili pepper (as dip sauce or as a recipe ingredient) |  |  |  |  |
| 62 |  | Lettuce |  |  |  |  |
| 63 |  | Celery (in salads, soups, main course) |  |  |  |  |
| 64 |  | Parsley (in salads, soups, main course) |  |  |  |  |
| 65 |  | Pumpkin |  |  |  |  |
| 66 |  | Chinese or green onion |  |  |  |  |
| 67 |  | Coriander (soups, main course) |  |  |  |  |
| 68 |  | Cucumber |  |  |  |  |
| 69 |  | Basil (soup, main course) |  |  |  |  |
| 70 |  | Radishes |  |  |  |  |
| 71 |  | Beet |  |  |  |  |
| 72 |  | Pore (soups, main course) |  |  |  |  |
| 73 |  | Rocoto (homemade sauce, as ingredient) |  |  |  |  |
| 74 |  | Broccoli |  |  |  |  |
| 75 | VEGETABLES | Snap Bean |  |  |  | Fill ONLY if Frequency answer is between 01 and 05:    01= Repeat answer (lunch + dinner)  02= No repetition |
| 76 |  | Caigua |  |  |  |  |
| 77 |  | Raw spinach |  |  |  |  |
| 78 |  | Cooked Spinach (Pesto spaguetti, other main courses, soup) |  |  |  |  |
| 79 |  | Huacatay (*Tagetes minuta* ) In ocopa sauce and other recipies) |  |  |  |  |

| 80 | CEREAL AND GRAINS | Rice |  | 01= Never  02= 1-2 times  03= 3-5 times  04= 6-9 times  05= 10-13 times  ----------------------  06= Once a day /  from 14 to 18 times  07= Twice a day /  from 19 to 28 times  08= 3 times a day /  from 29 to 42 times  09= 4 or more times a day /  From 43 to more times |  | Fill ONLY if Frequency answer is between 01 and 05:    01= Repeat answer (lunch + dinner)  02= No repetition |
| --- | --- | --- | --- | --- | --- | --- |
| 81 |  | Oatmeal, any brand |  |  |  |  |
| 82 |  | Barley (for breakfast, soft drinks) |  |  |  |  |
| 83 |  | 7 Seeds Mix breakfast |  |  |  |  |
| 84 |  | Thick Andean Corn "Choclo", Corn, Toasted corn kernals, Thick boiled corn kernals "mote" |  |  |  |  |
| 85 |  | Pop Corn |  |  |  |  |
| 86 |  | Wheat |  |  |  |  |
| 87 |  | Canihua *(Chenopodium pallidicaule)* |  |  |  |  |
| 88 |  | Amaranth |  |  |  |  |
| 89 |  | Quinoa |  |  |  |  |
| 90 | DAIRY | Whole Milk (Blue label “Gloria”, “Pura Vida”, “Ideal” or “Laive”, “Vaso de Leche” social program) |  |  |  |  |
| 91 |  | Light Milk (Red label “Gloria” “Ideal” or “Laive”, free lactose milk) (not soy) |  |  |  |  |
| 92 |  | Processed Soy Milk, any package. |  |  |  |  |
| 93 |  | Chocolate Milk |  |  |  |  |
| 94 |  | Condensed Milk |  |  |  |  |
| 95 |  | Milk Caramel |  |  |  |  |
| 96 |  | White Yogurt (natural or vanilla) |  |  |  |  |
| 97 |  | Fruit Yogurt, any flavor |  |  |  |  |
| 98 |  | Peruvian Fresh Cheese |  |  |  |  |
| 99 |  | Edam Chesse |  |  |  |  |
| 100 |  | Butter |  |  |  |  |

| 101 | TUBERS | Potato (Boiled, fried, stewed, etc) |  | 01= Never  02= 1-2 times  03= 3-5 times  04= 6-9 times  05= 10-13 times  ----------------------  06= Once a day /  from 14 to 18 times  07= Twice a day /  from 19 to 28 times  08= 3 times a day /  from 29 to 42 times  09= 4 or more times a day /  From 43 to more times |  | Fill ONLY if Frequency answer is between 01 and 05:    01= Repeat answer (lunch + dinner)  02= No repetition |
| --- | --- | --- | --- | --- | --- | --- |
| 102 |  | Chuño, Moraya (freeze-dried potatoes) |  |  |  |  |
| 103 |  | Olluco (*Ullucus tuberosus)* |  |  |  |  |
| 104 |  | Sweet Potato |  |  |  |  |
| 105 |  | Yucca |  |  |  |  |
| 106 |  | Maca (*Lepidium peruvianum*) |  |  |  |  |
| 107 |  | Tubers WITH their skin |  |  |  |  |
| 108 | BEANS AND PEAS | Lentils |  |  |  |  |
| 109 |  | Beans, all types |  |  |  |  |
| 110 |  | Dry habas |  |  |  |  |
| 111 |  | Fresh habas |  |  |  |  |
| 112 |  | Fresh Peas |  |  |  |  |
| 113 |  | Dry green peas |  |  |  |  |
| 114 |  | Non- processed Soy |  |  |  |  |
| 115 |  | Lima Beans, Chickpeas, Moron beans, Andean lupin |  |  |  |  |
| 116 | BAKERY | Baked French style Bread, Ciabatta, Integral, Andean style bread |  |  |  |  |
| 117 |  | Baked Yolk bread, Caracol, Coliza, Pizza bread, Hamburger or Hot dog bread, “Empanada” pastry (salty pastry usually with a bit of meat or chicken) |  |  |  |  |
| 118 |  | Bread from a mold |  |  |  |  |
| 119 |  | Pasta, any kind, Wantan |  |  |  |  |

| 120 | SWEET BAKERY | Chocolate cookies |  | 01= Never  02= 1-2 times 03= 3-5 times 04= 6-9 times 05= 10-13 times ----------------------  06= Once a day /  from 14 to 18 times  07= Twice a day /  from 19 to 28 times  08= 3 times a day /  from 29 to 42 times  09= 4 or more times a day /  From 43 to more times |  | Fill ONLY if Frequency answer is between 01 and 05:    01= Repeat answer (lunch + dinner)  02= No repetition |
| --- | --- | --- | --- | --- | --- | --- |
| 121 |  | Coockies, other flavors |  |  |  |  |
| 122 |  | Crackers (Soda, Integral, Vanilla, Margaret Chaplin) |  |  |  |  |
| 123 |  | Chocolate Cake, Pie or Cake |  |  |  |  |
| 124 |  | “Chancay” style cake (Cake made with yolk, baked twice and spiced with ginger), muffins, Cake, (any flavor but chocolate), Cupcakes, Donuts, Panettone. |  |  |  |  |
| 125 |  | “Alfajor”, “Pionono”, “Cachito”, “Mil hojas” (sweet cookie pastries with molasses sometimes stuffed with milk caramel)., Stuffed cake |  |  |  |  |
| 126 | CHOCOLATE | Cocoa |  |  |  |  |
| 127 |  | “Chocolate de taza” (Hot Chocolate beverage made from whole tablets of cacao with added sugar, cinnamon and clove). |  |  |  |  |
| 128 |  | Chocolate powdered flavoring mix (Milo, Nesquik, Kiwigen, etc.). |  |  |  |  |
| 129 |  | Chocolate bar (“Sublime”, “Princesa”, “lentejitas”, “triangulo”, etc.) |  |  |  |  |
| 130 |  | Stuffed chocolate (“Olé Olé”, “Beso de Moza”, etc.) |  |  |  |  |
| 131 | CANDY AND SWEETS | Brown Sugar |  |  |  |  |
| 132 |  | White Sugar |  | 01= Never  02= 1-2 times 03= 3-5 times 04= 6-9 times 05= 10-13 times ----------------------06= Once a day /  from 14 to 18 times  07= Twice a day /  from 19 to 28 times  08= 3 times a day /  from 29 to 42 times  09= 4 or more times a day /  From 43 to more times |  | Fill ONLY if Frequency answer is between 01 and 05:    01= Repeat answer (lunch + dinner)  02= No repetition |
| 133 |  | Hard, soft, toffee or gummy Candy, gum |  |  |  |  |
| 134 |  | Breakfast cereal, chocolate flavor (Angel, Kelloggs, etc.). |  |  |  |  |
| 135 |  | Breakfast cereal (angel, kellogs, etc.). |  |  |  |  |
| 136 |  | Jam, any flavor |  |  |  |  |
| 137 |  | Chocolate Wafer (Nick, Cua-Cua, Cañonazo, Morochas, etc.). |  |  |  |  |
| 138 |  | Wafer, other flavors (vanilla, strawberry) |  |  |  |  |
| 139 |  | Jelly, any flavor |  |  |  |  |
| 140 |  | Honey |  |  |  |  |
| 141 |  | Chocolate ice cream |  |  |  |  |
| 142 |  | Ice-cream |  |  |  |  |
| 143 |  | Popsicle |  |  |  |  |
| 144 | DRINKS AND BEVERAGES | Herbal Tea: chamomile, mint, Lemon Verbena, Cat's Claw, Eucalyptus, Horsetail, etc. |  | 01= Never  02= 1-2 times  03= 3-5 times  04= 6-9 times  05= 10-13 times  ----------------------  06= Once a day /  from 14 to 18 times  07= Twice a day /  from 19 to 28 times  08= 3 times a day /  from 29 to 42 times  09= 4 or more times a day /  From 43 to more times |  | Fill ONLY if Frequency answer is between 01 and 05:    01= Repeat answer (lunch + dinner)  02= No repetition |
| 145 |  | “Chicha Morada” (traditional non-alcoholic beverage made with boiled red corn and often with bits of pineapple skin). |  |  |  |  |
| 146 |  | Natural lemonade, other beverages with lemon |  |  |  |  |
| 147 |  | Tea |  |  |  |  |
| 148 |  | Coffee |  |  |  |  |
| 149 |  | Artificially flavored Juice (“frugos”, “pulpín”, etc.). |  |  |  |  |
| 150 |  | Soda powder (Tang, Universal, Artificial Purple “chicha morada” flavor, etc.) |  |  |  |  |
| 151 |  | Citrus Punch (Cifrut, Tampico, etc.) |  |  |  |  |
| 152 |  | No colored soda (Sprite, 7up, etc.) |  |  |  |  |
| 153 |  | Colored Soda (Pepsi, Kola Real, Inca Kola, Oro, Coca Cola, Fanta, Kola Inglesa) |  |  |  |  |
| 154 |  | Sports drink (Powerade, Gatorade, Sporade) |  |  |  |  |
| 155 |  | Energy Drink (Maltin Power, Red Bull, etc.). |  |  |  |  |
| 156 | SNACKS | Chips, Banana chips, Sweet potato chips,, Doritos, Cuates, Chizitos, Piqueo Snack, Cheese Tris, etc.) |  |  |  |  |
| 157 | SPICES | Cinnamon, Clove |  | 01= Never  02= 1-2 times  03= 3-5 times  04= 6-9 times  05= 10-13 times  ----------------------  06= Once a day /  from 14 to 18 times  07= Twice a day /  from 19 to 28 times  08= 3 times a day /  from 29 to 42 times  09= 4 or more times a day /  From 43 to more times |  | Fill ONLY if Frequency answer is between 01 and 05:    01= Repeat answer (lunch + dinner)  02= No repetition |
| 158 |  | Pepper, Cumin |  |  |  |  |
| 159 |  | Aji-no-moto (Monosodium glutamate) |  |  |  |  |
| 160 |  | Soy Sauce |  |  |  |  |
| 161 |  | Artificial seasonings (Sybarite, Mrs. Gusta, Maggi seasoning cube) |  |  |  |  |
| 162 |  | Palillo (turmeric-like seasoning), Oregano, Laurel |  |  |  |  |
| 163 |  | Achiote (Annatto seed) |  |  |  |  |
| 164 |  | Ginger |  |  |  |  |
| 165 | PROCESSED FOOD | Tomato Sauce (canned, paste or powder), Pomarola tomato sauce |  |  |  |  |
| 166 |  | Mayonnaise, Mustard, Ketchup, Processed Chili, “Rocoto” chili (*Capsicum pubescens*), “Huancaína” Sauce |  |  |  |  |
| 167 |  | Instant soup, mashed potatoes, breaded mix |  |  |  |  |
| 168 | OTHER | Salt |  |  |  |  |
| 169 |  | Egg |  |  |  |  |
| 170 |  | Vinegar |  |  |  |  |
| 171 |  | Linseed (seed, in bread, emoliente, etc.) |  |  |  |  |
| 172 |  | Mushrooms (as ingredient with Laurel), Mushrooms |  |  |  |  |
| 173 |  | Yuyo (ceviches, seafood and soups) |  |  |  |  |
| 174 |  | “Tamal” (sweet and salty corn snack cooked in a plantain leaf, usually with a bit of chicken). |  |  |  |  |
